# New Variant of Porcine Epidemic Diarrhea Virus, United States, 2014

**DOI:** 10.3201/eid2005.140195

**Published:** 2014-05

**Authors:** Leyi Wang, Beverly Byrum, Yan Zhang

**Affiliations:** Ohio Department of Agriculture Reynoldsburg, Ohio, USA

**Keywords:** Porcine epidemic diarrhea virus, variant, phylogenetic analysis, United States, PEDV, OH851, viruses

**To the Editor:** Porcine epidemic diarrhea (PED) was first reported in the United Kingdom in 1971 (*1*). The disease was characterized by severe enteritis, vomiting, watery diarrhea, dehydration, and a high mortality rate among swine. Subsequently, the causative agent of PED was identified as porcine epidemic diarrhea virus (PEDV), which belongs to the family *Coronaviridae* (*2*) and contains an enveloped, single-stranded positive-sense RNA genome. PEDV has been reported in many other countries, including Germany, France, Switzerland, Hungary, Italy, China, South Korea, Thailand, and Vietnam (*3*) and was first identified in the United States in May 2013. By the end of January of 2014, the outbreak had occurred in 23 US states, where 2,692 confirmed cases (www.aasv.org/news/story.php?id = 6989) caused severe economic losses. Recent studies have shown that all PEDV strains in the United States are clustered together in 1 clade within the subgenogroup 2a and are closely related to a strain from China, AH2012 (*4*,*5*).

In the state of Ohio, the first PED case was identified in June of 2013; since then, hundreds of cases have been confirmed by the Animal Disease Diagnostic Laboratory of the Ohio Department of Agriculture. In January of 2014, samples from pigs with unique disease, suspected to be PED, were submitted to this laboratory. Sows were known to be infected, but piglets showed minimal to no clinical signs and no piglets had died. 

According to real-time reverse transcription PCR, all samples from the piglets were positive for PEDV. Subsequently, the full-length genome sequence of PEDV (OH851) was determined by using 19 pairs of oligonucleotide primers designed from alignments of the available genomes from PEDVs in the United States (*6*,*7*). On the basis of BLAST (http:blast.ncbi.nlm.nih.gov/Blast.cgi) searches, strain OH851 showed 99% and 97% nt identity to PEDVs currently circulating in the United States (Colorado, Iowa, Indiana, Minnesota) for the whole genome and the full-length spike (S) gene, respectively. By distinct contrast, strain OH851 showed only 89% or even lower nucleotide identity to PEDVs currently circulating in the United States in the first 1,170 nt of the S1 region. In that region, nucleotide similarity to that of a PEDV strain from China (CH/HBQX/10, JS120103) was 99%, suggesting that strain OH851 is a new PEDV variant. Phylogenetic analysis of the complete genome indicated that the novel OH851 PEDV is clustered with other strains of PEDV currently circulating in United States, including another stain from Ohio, OH1414 (Figure, panel A). However, phylogenetic analysis of the full-length S gene showed that strain OH851 is clustered with other strains of PEDV from China and most closely related to a PEDV strain from China, CH/HBQX/10 (*8*), but distantly related to other PEDV strains currently circulating in the United States and strain AH2012 (Figure, panel B). This finding strongly suggests that strain OH851 is a variant PEDV. In comparison with the S gene of other strains from the United States, the S gene of strain OH851 has 3 deletions (a 1-nt deletion at position 167, a 11-nt deletion at position 176, and a 3-nt deletion at position 416), a 6-nt insertion between positions 474 and 475, and several mutations mainly located in the first 1,170 nt of the S1 region. 

**Figure:**
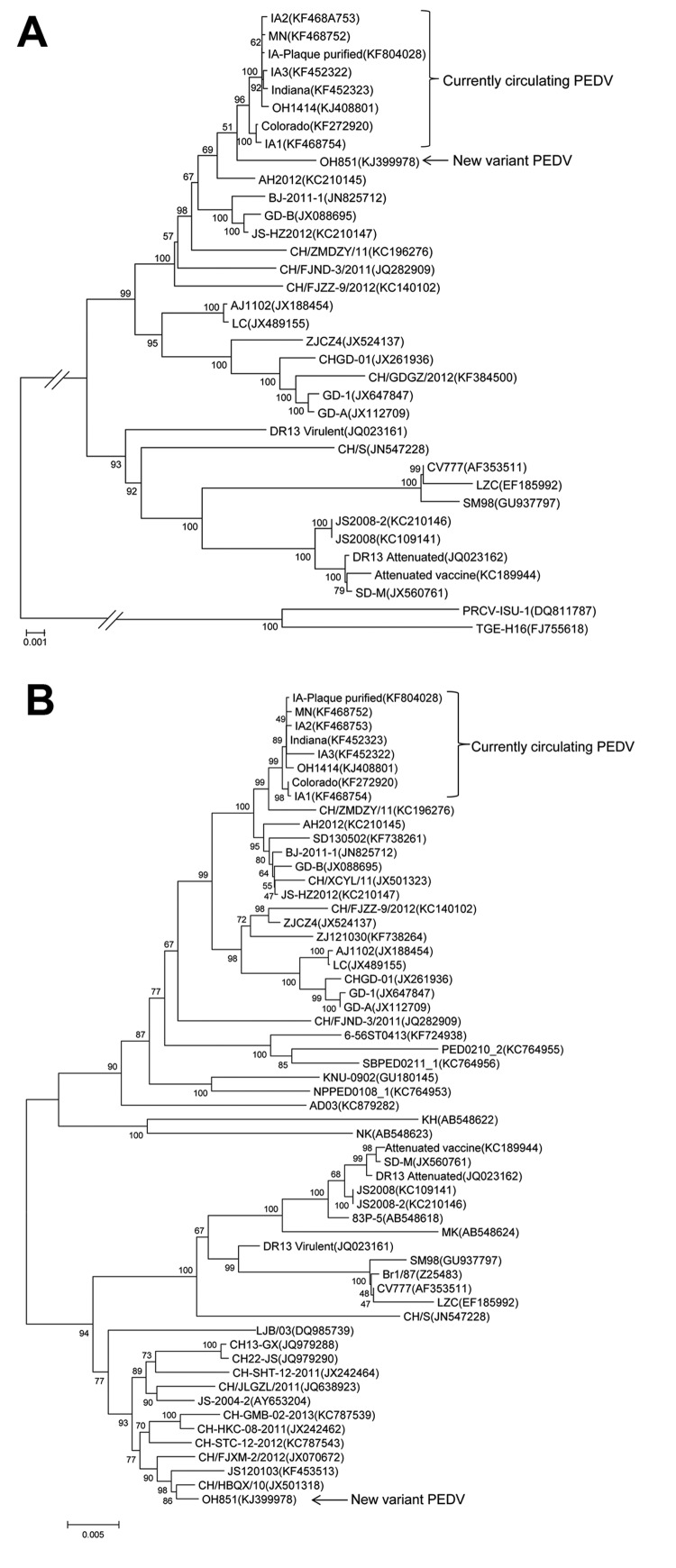
Phylogenetic tree of the whole-genome sequences of 33 strains of porcine epidemic diarrhea virus (PEDV) (A) and of spike protein nucleotide sequences of 56 strains of PEDV (B), including the new variant PEDV (OH851) and 8 PEDV strains currently circulating in the United States. The dendrogram was constructed by using the neighbor-joining method in MEGA version 6.05 (www.megasoftware.net). Bootstrap resampling (1,000 replications) was performed, and bootstrap values are indicated for each node. Reference sequences obtained from GenBank are indicated by strain name and accession number. Scale bars indicate nucleotide substitutions per site.

It is highly possible that the sequence deletions, insertion, and mutations found in variant strain OH851 might have contributed to the reduced severity of the clinical disease in the piglets. More animal studies are needed to test this hypothesis. The unique deletion and insertion feature also represents a target for diagnostic assays to differentiate between currently circulating PEDV strains and new variants.

The low nucleotide identity in the 5′-end S1 region (first 1,170 nt) region and high nucleotide identity in the non-5′-end S1 region of the variant strain, compared with that of the PEDVs currently circulating in the United States, suggest that this new PEDV variant might have evolved from a recombinant event involving a strain from China. Because the new variant does not cause severe clinical disease, including death, the novel virus is a potential vaccine candidate that could protect the US swine industry from the infection caused by the virulent strain of PEDV currently circulating in United States.
